# Be brave, look for meaning: highlights of the tenth annual meeting of the National Cancer Research Institute

**DOI:** 10.3332/ecancer.2015.500

**Published:** 2015-01-12

**Authors:** Audrey Nailor, Sioned Pearce, Ian Lewis

**Affiliations:** 1 Cancer Intelligence Ltd, Bristol BS6 5RL, UK; 2 Cardiff University, WISERD Education, Cardiff School of Social Sciences, 46 Park Place, Cardiff CF10 3BB, UK; 3 Department of Research, Tenovus, 9th Floor Gleider House, Ty Glas Road, Cardiff CF14 5BD, UK

**Keywords:** end-of-life care, cancer communication, cancer statistics, circulating biomarkers, cancer evolution

## Abstract

The tenth Annual Meeting of the National Cancer Research Institute (NCRI) conference took place in Liverpool, UK. Just under 2000 delegates were estimated to have attended the conference, predominantly from the UK and Europe.

It was a multidisciplinary gathering aimed at cancer professionals at every level. The conference included primers on basic science and public communication as well as workshops on more advanced topics.The conference was grouped into six main themes, which this report will address in greater detail.

## Introduction

The tenth annual meeting of the National Cancer Research Institute (NCRI) took place in Liverpool, UK. The conference featured 179 speakers from around the world, roughly one-quarter of these were females, and it was attended by a estimate of under 2000 delegates. The majority of the attendees came from the UK and Europe.

It was a multidisciplinary gathering aimed at cancer professionals at every level, the conference was centred on six broad themes, ranging from end-of-life care to tumour heterogeneity, which this report will discuss in a greater detail. Professor Richard Marais, chair of the 2014 Scientific Committee, urged attendees to ‘be brave’ and attend sessions outside of their usual realm of practice.

## Theme: information, patients and the public

Communication-centred presentations and workshops stressed the importance of making cancer research accessible to patients and the public.

Kat Arney, Cancer Research UK Science Information manager, launched the programme on Sunday with an informative talk on ‘Hitting the Headlines’, discussing motivations, processes and outcomes for media engagement to promote scientific findings and publications. The session was a practical and straightforward introduction to achieving accurate scientific coverage in the media, pitched at public relation (PR) or marketing professionals and scientists. More coverage on communicating psychosocial scientific research may have benefited attendees from charities and organisations delivering and evaluating support for people living with cancer.

Professor Sir David Spiegelhalter of the University of Cambridge, UK, gave an inspiring presentation on the pitfalls of misinterpretation in communicating scientific statistics. He emphasised the impact of ‘framing’ numbers and statistics in a positive or negative light, and stressed how framing can lead to bias, which can impact behaviour. Spiegelhalter notes that framing statistics in ‘natural frequencies’ encourages understanding and limits bias.

In particular, both speakers argued that ‘numbers aren’t helpful’—laypeople prefer natural comparisons to statistics. Spiegelhalter noted that cancer patients are more likely to understand phrases like ‘one in 100 patients will have side effects’ than a detailed statistical information, and will behave accordingly. Arney pointed out that visualisations and metaphors have a greater impact on public understanding.

These results can impact the behaviour of everyone, from cancer patients to policymakers, thus emphasising the importance of clear cancer communication.

## Theme: survivorship and end-of-life care

The theme of ‘survivorship and end-of-life care’ highlighted the current debates and findings on scientific and psychosocial issues in cancer research. Often neglected from a scientific perspective, these sessions provided useful information about what happens at the end of the cancer story. This reflects the broad themes of this year’s NCRI conference, in which, unusually, a palliative care session was selected as a plenary lecture.

Professor William Breitbart of the Memorial Sloan Kettering Cancer Centre, USA, focused on an area of need that arises for many cancer patients facing terminal diagnosis—the loss of meaning. ‘Loss of meaning is not druggable,’ he stated.

His presentation explored the use of existentialism in palliative care for people with cancer, covering such concepts as despair, meaning, spirituality, legacy, creativity, and experiential existence at the end of life.

He also described randomised controlled trials that showed that loss of meaning can be mitigated against or even reversed with his novel counselling intervention, the results of which showed that meaning can buffer against the effects of pain and fatigue and even lower the incidence of depressive symptoms. In fact, Breitbart showed that helping cancer patients find meaning and ‘turn tragedy into personal triumph’ is more effective at reducing psychological symptoms than some drugs available—and a greater emphasis should be placed on this type of intervention [1].

Another aspect of the role of primary care in cancer treatment is the ongoing support of people who have already experienced a cancer diagnosis and require follow-up and care in their communities.

Dr Fiona Walter of the University of Cambridge, UK, discussed the role of primary care in the UK [2]. She described that in the UK there is a system in which general practitoners (GPs) carry out regular ‘Cancer Care Reviews’ with their patients as part of their ongoing follow-up following a cancer diagnosis. Whilst the majority of GPs reported that they did carry out these reviews, they appeared to be done mostly on an ad-hoc way with very few adhering to a common reporting structure. This raises concerns: not only does it undermine confidence in standardised quality control, but it represents a missed opportunity to collect valuable data identifying potential un-met needs for cancer patients in primary care.

## Theme: diagnosis and therapy

The broad theme of diagnosis and therapy included sessions on biomarkers, screening, and treatment.

Under this theme Professor Lewis Cantley, Director of the Cancer Centre at Weill Cornell Medical College, presented key milestones and events in the testing and elucidation of the PI3k lipid kinase pathway, which his group discovered in the mid-1980s [3].

PI3K plays a major role in the growth of all human tissues, but overactivation of PI3K, or inactivating mutations in phosphatase and tensin homolog (PTEN), are among the most common events in solid tumours. The importance of this pathway is highlighted by the fact that there are more than 30 drugs currently in clinical trials that target PI3K and its associated pathway. The next issue is to determine now is whether patients are most likely to benefit from treatment with PI3K inhibitors as single agent drugs or in combination with other drugs.

Professor Cantley ended the presentation with a summary of his preliminary work in which PI3K inhibition when supplemented with a poly ADP ribose polymerase (PARP) inhibitor, lead to a dramatic reduction of tumour load in mouse models of triple negative breast cancer. Professor Cantley and his team are now testing this drug combination in early stage clinical trials in women with triple negative breast cancer and ovarian cancer.

Approaching the role of PTEN deficiencies from another angle, Professor Kevin Prise of the Queen’s University Belfast, UK, noted that loss of PTEN function has been reported to confer resistance to radiation treatment. He presented a proffered paper on combining targeted radiotherapy with DNA damage modulators in PTEN-inducible castration-resistant prostate cancer [4]. His research in mouse models has suggested that an increased sensitivity to radiation treatment may be inducible with this combined treatment, with a reported median survival extended from 27 days to 88 days with no significant toxicity.

Biomarkers as agents of cancer screening were another frequently mentioned topic. Replacing solid-tissue biopsies with blood samples—or ‘liquid’ biopsies—was a particular trend of discussion, as the circulating biomarkers have now become increasingly valuable in diagnostic techniques. In an introductory primer on immunotherapy for researchers from other fields, Professor Peter Johnson of Cancer Research UK stressed the need for accurate biomarkers.

‘What can we learn from circulating tumour cells in lung cancer’? asked Professor Caroline Dive, of the Cancer Research UK Manchester Institute [5]. Circulating tumour cells (CTCs) serve as diagnostic biomarkers in small-cell lung cancer, which is not a good target for traditional biopsies, but have unclear clinical utility in non-small-cell lung cancer. CTCs are also useful for genome analysis, as the cell contains the genomic information of the host tumour. Dive discussed her research group’s experiments in CTC explants, in which circulating tumour cells are removed from individual patients and placed in mouse models, where they may provide information on drug targets and drug resistance mechanisms.

‘Five years from now, blood tests will stratify cancer patients’, Dive predicted. ‘Blood tests will predict relapses and personalise medicine’.

## Theme: epidemiology and prevention

Sessions given under this theme discussed the broader contexts of cancer medicine, including screening and clinical trials.

‘Far from being dead, dying or impotent, clinical trials are alive, well and potent,’ said Professor Andrew Hughes of AstraZeneca and the University of Manchester. Hughes presented a retrospective look at the past decade’s development of clinical trials and his expectations for the future in a celebratory lecture entitled ‘Whither clinical trials’?

Ten years ago, 23 patients had to be enrolled and screened to obtain one eligible patient for a clinical trial. Surprisingly, the same is true today–but Hughes stated that new innovations such as virtual biopsies, smartphone technology, and targeted trials will decrease this waste and cost.

Virtual biopsies were certainly a trendy topic at this year’s NCRI, but Hughes suggested that they might play a serious role in reducing clinical reliance on solid tissue biopsies. Solid biopsies are unrepresentative of tumour heterogeneity, and may be unreliable in screening patients for clinical trials, resulting in more patients being rejected for appropriate trials.

Hughes highlighted smart electronic data capture as another way to streamline clinical trials, with an emphasis on smartphones. Instead of relying on laborious data collection processes that delay results and may inconvenience the patient, Hughes suggested that patients could use smartphones and cloud technology to log their own details on specialised apps. Advantages cited included fewer delays, more opportunities to adapt ongoing trials based on emerging insights, and retention of each patient’s unique ‘voice’.

Finally, Hughes predicted the continuing rise of the bucket/basket trial model, stating that the field should move from huge ‘carpet-bombing’ clinical trials to more specific and streamlined entities defined by genomic profiling or biomarkers. The challenges of this move include the difficulties of forming a consortia-based approach from multiple institutions, regulatory difficulties, and pinning down the currently nebulous definition of biomarkers.

Professor Peter Sasieni of the University of London, UK, hosted a session on cancer prevention through screening programmes, aspirin use, and obesity control. Professor Jack Cuzick of the Wolfson Institute, London, UK, Professor Peter Rothwellof Oxford University, Oxford, UK, and Dr Tom Lobstein of the World Obesity Federation, London, UK, discussed the context and detail of these approaches to prevention and the importance of progressing this important, and often neglected, area of cancer research.

Analysis on breast cancer screening showed relative risk against ten-year absolute risk for 50-year-old women attending screening, and it affects the high-risk group numbers overall. The story of 40 years’ research and meta-analysis into aspirin as a cancer preventative shows success in a number of trials and for all cancers. Finally, reducing obesity to lower cancer incidence was posed as a challenging task given the imbalance in funding for unhealthy food promotion against government health promotions.

## Theme: health services research

A debate hosted by Professor Malcolm Mason of Cardiff University, UK, titled ‘This house believes that the Cancer Drugs Fund has been good for British cancer patients’ was by far the most popular session under this theme.

The debate delivered on its provocative title through well-argued confirmations and critiques from both sides of the argument. The premise of the question was immediately put into refute by a division in speakers according to their representation of the four separate nations of the UK.

Dr Martin Eatock of the Belfast Health and Social Care Trust, Belfast, UK, argued against the benefits of the Cancer Drugs Fund (CDF) from the Northern Ireland perspective, highlighting the difference between easy access to drugs in principle and in practice, even as in the case of individual funding requests.

Dr Tom Crosby of the Velindre Cancer Centre, Cardiff, UK, discussed the fact that the CDF does not apply in Wales, given the fact that NICE overrides technological assessment system and the existence of the all Wales Strategy Group.

Professor Peter Clark, Head of the Cancer Drugs Fund, argued for the CDF from an English perspective. Finally, Professor David Cameron of the Edinburgh Cancer Research Centre, Edinburgh, UK, gave a strong ‘for’ argument after outlining the Scottish perspective, particularly the disparity between NICE systems of allocation and those that of the CDF.

Discussion highlighted disparities rooted in devolution and response to audience’s questions and patient’s experiences at the receiving end of the fund. Strong arguments for retaining the NICE allocation of drugs funds for more equitable, cost effective, and transparent allocation were contested with statements around quicker access and a more robust peer review process through the cancer drugs fund. The history and rationale behind the creation of the CDF also featured. The ‘against’ side argued that establishment of the fund in 2010 exposed the policy gap between process and reality while the ‘for’ side responded by detailing repercussions.

Following the opening remarks, there was an even spread between the number of people who agreed and disagreed with the premise that ‘the Cancer Drugs Fund has been good for British cancer patients’.

However, it is worth noting that some people did take issue with the title of the debate, as the CDF has only granted benefits to those in England who could access it. Even with that taken into account, there was still a very sizeable shift by the end of the debate, with around 75% of the hall disagreeing with the assertion that the CDF was a good thing.

Taking into account the calibre and breadth of expertise that attended the NCRI conference, this is a notable outcome belying the fact that there has not been a similar size groundswell of opinion against the CDF amongst experts and professionals in the media.

Another topic of discussion in this theme was the ‘gatekeeper’ healthcare system in the UK, which renders the role of the GP in diagnosing cancer, or at least identifying symptoms that require further investigation, absolutely crucial. Also notable is the key interaction between primary care and secondary care, particularly when it comes to accessing the appropriate diagnostics required to confirm or rule out a diagnosis of cancer.

Shifting the focus from the UK, Professor Peter Vedsted of Aarhus University, Denmark, explored existing models of healthcare systems in other regions. He described his involvement in the Danish healthcare system, how now with improved speed the patients with a suspected cancer can access diagnosis and prognosis through service redesign. This aspect drew particular envy from one questioner, who said that these described systems, joined-up service design, and supporting technology would be appropriate for use in the UK.

## Theme: the cancer cell and model systems

Tumour heterogeneity was another theme, with researchers acknowledging that solid-tissue biopsies are not indicative of the complexity of tumours, either within the same tumour or across the biological system.

In a seminar on cancer evolution, Dr Scott Carter of the Broad Institute of MIT–Harvard, Cambridge, USA, presented his work on computational dissection of tumours to trace the origins of their heterogeneity and reconstruct their phylogeny. Computational dissection—predicting the genomic characters of tumours through modelling rather than physical biopsies—could resolve some of these issues of representation.

Nicholas McGranahan of Cancer Research UK, London Research Institute, London, UK, supported these findings in his presentation on lung cancer evolution [7]. Examining seven cases of non-small-cell lung cancer (NSCLC) from various donors–including non-smokers, smokers, and ex-smokers– McGranahan found evidence for branched tumour evolution and a variety of driver mutations in all cases. Understanding this heterogeneity and variety of evolutionary divergence may provide key molecular underpinnings to treat this aggressive disease.

The Cancer Research UK (CRUK) Lifetime Achievement Award lecture is one of the highlights of the NCRI conference and this year was no exception. Professor Ron Laskey of the University of Cambridge, UK, is a scientist who has achieved success in areas that include the control of DNA synthesis, the transport of macromolecules between the nucleus and cytoplasm, and a musical career that has led to two albums including ‘Songs for Cynical Scientists’. Sadly, only the first two aspects were on display in his presentation at this year’s conference. However, what he did show was that work carried out decades previously still has far-reaching implications to cancer research and treatment today, and also that cancer research is a huge collaborative effort as he paid tribute to those who inspired him, the collaborations he formed, and the researchers whom he trained.

## Conclusion

The six themes provided good coverage on key areas of cancer research, in which communication and palliative care were given equal standing supported by biological and clinical results.

In general, this was a satisfying meeting of NCRI, covering a broad range of topics. More representation from female professionals would have provided a good balance; but in other aspects, we see NCRI is moving forward with its crowd-sourced amenities, such as a live Twitter feed and suggestion boards. It provided an interesting platform for informed debate on newsworthy topics such as the CDF, and actively encouraged multidisciplinary interactions. Hopefully, the future NCRI meetings will build on these positive strategies.
